# 3D printing of microneedle arrays for hair regeneration in a controllable region

**DOI:** 10.1186/s43556-022-00102-2

**Published:** 2023-01-05

**Authors:** Rong Li, Xin Yuan, Li Zhang, Xuebing Jiang, Li Li, Yi Zhang, Linghong Guo, Xide Dai, Hao Cheng, Xian Jiang, Maling Gou

**Affiliations:** 1 State Key Laboratory of Biotherapy and Cancer Center, West China Hospital, Sichuan University, 610041 Chengdu, China; 2 Department of Plastic and Burn Surgery, West China Hospital, Sichuan University, 610041 Chengdu, China; 3 Department of Dermatology, West China Hospital, Sichuan University, 610041 Chengdu, China; 4 Laboratory of Dermatology, Clinical Institute of Inflammation and Immunology (CIII), Frontiers Science Center for Disease-related Molecular Network, West China Hospital, Sichuan University, 610041 Chengdu, China; 5 Huahang Microcreate Technology Co., Ltd, 610042 Chengdu, China

**Keywords:** Microneedles, Hair regeneration, Personalized treatment, 3D printing, Regenerative medicine

## Abstract

Hair loss is a common skin disease that causes intense emotional suffering. Hair regeneration in a personalized area is highly desirable for patients with different balding conditions. However, the existing pharmaceutical treatments have difficulty precisely regenerating hair in a desired area. Here, we show a method to precisely control the hair regeneration using customized microneedle arrays (MNAs). The MNA with a customized shape is fast fabricated by a static optical projection lithography process in seconds, which is a 3D printing technology developed by our group. In the mouse model, MNA treatment could induce hair regrowth in a defined area corresponding to the customized shape of MNA. And the regenerated hair promoted by MNAs had improved quality. Cellular and molecular analysis indicated that MNA treatment could recruit macrophages in situ and then initiate the proliferation of hair follicle stem cells, thereby improving hair regeneration. Meanwhile, the activation of the Wnt/β-catenin signaling pathway was observed in hair follicles. The expressions of *Hgf*, *Igf 1* and *Tnf-α* were also upregulated in the treated skin, which may also be beneficial for the MNA-induced hair regeneration. This study provides a strategy to precisely control hair regeneration using customized microneedle arrays by recruiting macrophages in situ, which holds the promise for the personalized treatment of hair loss.

**Supplementary Information:**

The online version contains supplementary material available at 10.1186/s43556-022-00102-2.

## Introduction

Hair loss is a common skin disease that impacts the body image, social interactions, and psycho-emotional health [1, 2]. In clinical practice, alopecia is characterized by a range of patient circumstances and needs, involving different sites, degrees of severity and etiologies [3, 4]. Although various medical treatments (e.g., minoxidil (MXD), finasteride, etc.) are effective in certain patients, they have not reached the desired effect for precise hair regeneration. Therefore, how to precisely control hair regeneration remains a challenge.

Microneedle arrays (MNAs) are micron-scale clusters of needles used for minimally invasive and painless puncture of the skin. In addition to efficient drug delivery, they have been widely used to regulate the dermal microenvironment and promote tissue regeneration via mechanical stimulation and microwounding. For example, microneedles alone have been used for the treatment of acne [5], keloids [6], wrinkles [7], etc. Regarding hair loss treatment, some studies have shown that drug-free microneedles can remodel the perifollicular microenvironment in the balding region and then induce hair regeneration [8, 9]. In addition, the specific distribution of microneedles can deliver substances to specific locations [10, 11]. Based on these reports, we propose a hypothesis that the customized MNA can locally modulate the dermal microenvironment and then precisely promote the hair regeneration in situ.

3D printing can be used for the flexible customization of MNA with a fine structure and personalized shape [12, 13]. Recently, our group developed a 3D printing technology, static optical projection lithography (SOPL), for flexibly customizing MNA, which can rapidly construct customized MNA within seconds through the spatial polymerization of monomer solution induced by static projected digital light [11, 14]. Here, SOPL technology was used to quickly fabricate MNAs with designed shapes. Next, we investigated the feasibility of customized MNA to induce precise hair regeneration in situ. It was found that the treatment of round-shaped MNA on the dorsal skin of mice induced early hair regeneration with a round shape. The mechanisms were found that MNA treatment could recruit macrophages in situ and then initiate the proliferation of hair follicle stem cells, thus improving hair regeneration. In addition, activation of β-catenin was also observed in the treated hair follicles, which may involve *Wnt 10a* and *Lef 1* (a nuclear responder of Wnt signals). The upregulation of hepatocyte growth factor (*Hgf*), insulin-like growth factor 1 (*Igf-1*) and tumor necrosis factor-α (*Tnf-α*) was recognized in the MNA-treated area, which may also be beneficial for the MNA-induced hair regeneration. This study provides a new strategy for improving hair regeneration in situ and points to a new direction for future regenerative medicine.

## Results

### Customization of MNA by SOPL

**Fig. 1:**
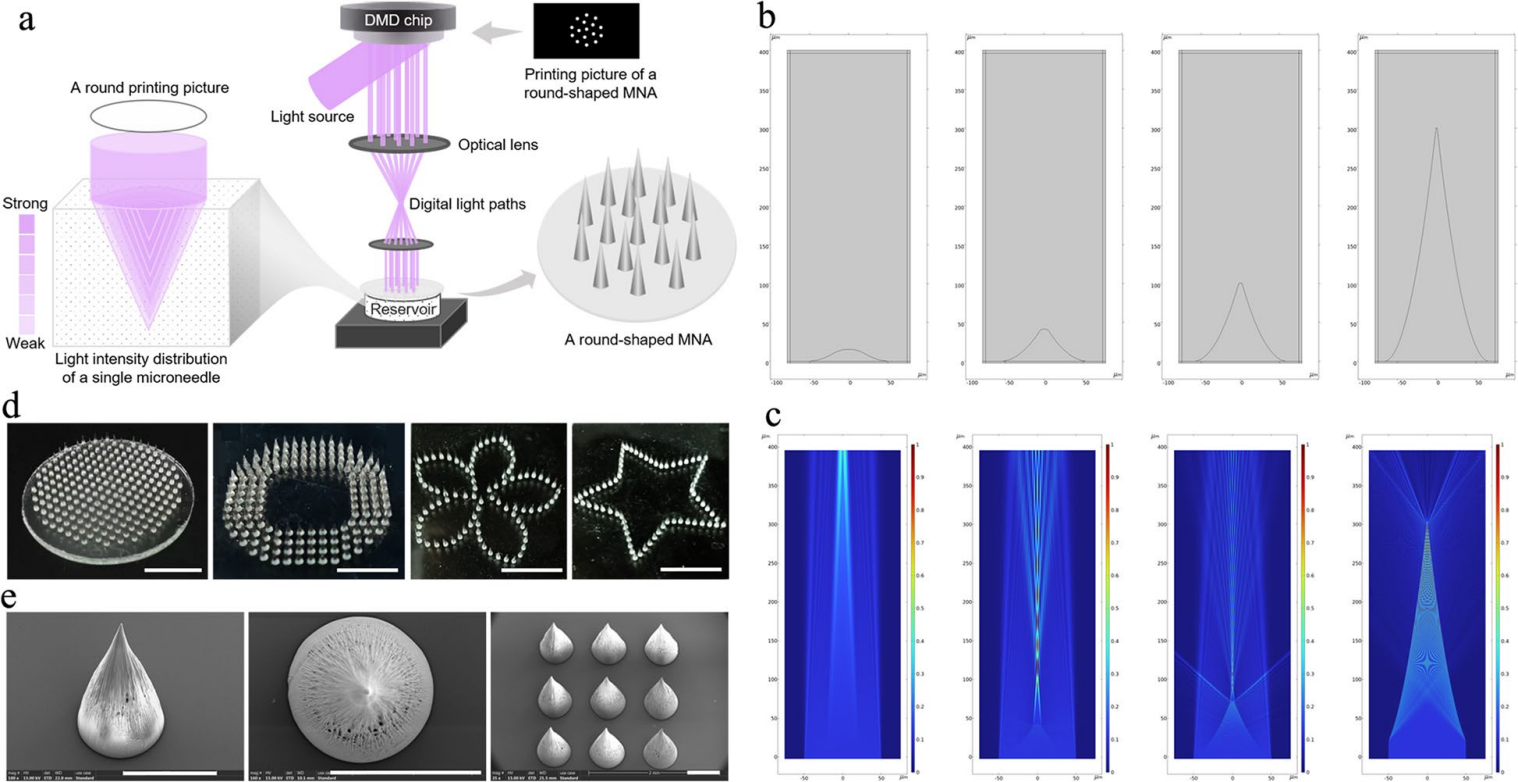
Customization of MNA by SOPL. **a** Schematic diagram of the principle for customization of the MNA by SOPL. **b** 2D geometric models of a microneedle at heights of 10, 40, 100 and 300 μm constructed in COMSOL. **c** The light intensity distribution of the microneedle at heights of 10, 40, 100 and 300 μm simulated by COMSOL. **d** Photographs of round-, annular-, petaloid-, and pentagonal star-shaped MNAs. (Scale bars: 500 mm). **e** SEM images of the microneedles and the MNAs. (Scale bars: 500 μm)

To quickly fabricate the customized MNA for precise hair regeneration, a SOPL technology was employed in this study [11]. The scheme of the SOPL technology for customizing the MNA is presented in Fig. 1a. As shown in Fig. 1a, MNA formed through the spatial polymerization of monomer solution, which is controlled by the specific spatial distribution of light intensity. The light intensity distribution of a microneedle in the photosensitive resin (Ausbond, A371) can be described as the point diffusion function, which is similar to the Gaussian distribution [15], wherein the light intensity gradually weakened from the center of the focal plane to the outside. When digital light penetrates the photosensitive resin, light is absorbed with the gradual increase in penetration depth, resulting in the gradual decrease in light intensity [16]. Accordingly, the specific spatial distribution of light intensity allows the photosensitive resin to precisely polymerize to form a microneedle. During the manufacturing process, a light beam was modulated into a customized pattern by a digital micromirror device (DMD) and projected to induce the spatial polymerization of the monomer to form the MNA.

To visualize the formation process of microneedle in the SOPL technology, we simulated the light propagation and light intensity distribution during microneedle fabrication using finite element analysis. A simplified 2D geometric model with two parabolic edges imitating the microneedle at designed height (*H*) was constructed in COMSOL software (Fig. 1b). As seen from the numerical simulation in Fig. 1c, at the beginning of light irradiation, the self-aligned lens effects were produced due to the polymerization of monomer solution [17]. Then the light converged to the center, and the monomer solution was polymerized to form the microneedle as a consequence of the light intensity distribution of the conical shape. The simulation results implied that the microneedle is formed in the manner of growth, and the spatial distribution of light intensity can be the primary factor enabling the fabrication of the microneedle via SOPL technology. This demonstrated that the ability of SOPL technology to rapidly customize high-quality microneedles within 3 s.

By designing printing pictures, the SOPL technology could prepare customized MNA with various shapes, such as round-, annular-, petaloid-, and pentagonal star-shaped MNAs (Fig. 1d). The scanning electron microscope (SEM) images showed the morphologies of the microneedles and the MNAs (Fig. 1e). The obtained MNAs did not show the layer-by-layer structure present in the common 3D-printed products, and could be fast customized within 3 s. Therefore, SOPL technology provides technical support for manufacturing customized MNAs, which are then used for precise hair regeneration.

### Characterization of MNA

**Fig. 2:**
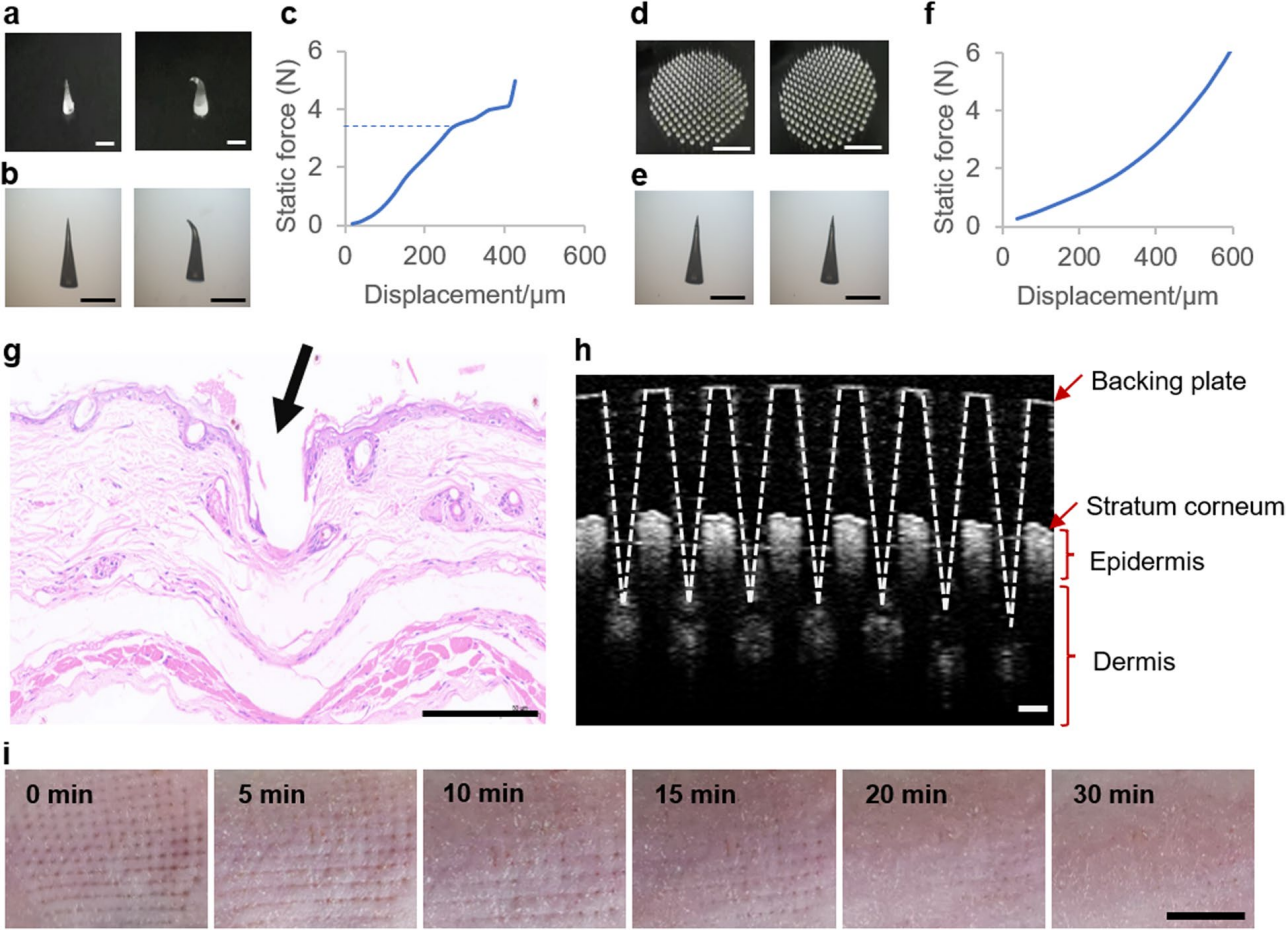
Characterization of the MNA. Pictures of microneedle taken with cameras **a** and microscopes **b** showed that the tip of the microneedle was bent after compression tests. (Scale bars: 500 μm). **c** Force-displacement curve of a single MN. The largest deformation occurred at approximately 3.3 N compression force. **d** The camera pictures of the MNA indicated no obvious change after compression tests. (Scale bar: 500 mm). **e** Microscopic images of one needle from the array showed no obvious difference between before and after compression tests. (Scale bar: 500 μm). **f** The force-displacement curve of the MNA showed no obvious turning point. H&E-stained cross-section **g** and OCT **h** revealed that the MNA could penetrate the epidermal layer of the skin. (Scale bars: 200 μm). **i** Skin pictures recorded the quick skin healing after the treatment of the round-shaped MNA. (Scale bar: 500 mm)

To characterize the performance of MNA for treating hair loss, the mechanical properties, puncture performance, and skin healing were assessed. The mechanical strength of a single microneedle and MNA were tested by compression tests. The pictures of one microneedle before and after compression demonstrated that the tip of the microneedle was bent after compression (Fig. 2a and b). The force-displacement curve was almost linear at the beginning, then the microneedle bent and a turning point appeared on the curve, at which the compression distance was approximately 280 μm and the compression force was approximately 3.3 N (Fig. 2c). The pictures of the MNA before and after compression (Fig. 2d and e) showed that there was no obvious change in the microneedle after compression, and the force-displacement curve was almost a smooth line (Fig. 2f). The differences between Fig. 2b and e were possibly because each needle in the MNA received less pressure than a single microneedle. An insertion force of 0.1–3 N has been reported to be sufficient to manually insert the microneedle into the skin [18]. This indicates that the MNAs are mechanically strong enough for skin puncture. Hematoxylin-eosin (H&E) staining of skin tissue after MNA puncture indeed showed that the MNA penetrated the epidermal layer (Fig. 2 g). In addition, optical coherence tomography (OCT) demonstrated that the MNA punctured into the superficial dermis (Fig. 2 h). Therefore, MNAs fabricated by SOPL can be used for skin puncture. After confirming the capacity of the MNA for minimally invasive skin puncture, the round-shaped MNA was applied to the mouse skin to assess the healing of the skin. After the MNA treatment, visible micropore arrays corresponding to the shape of MNA formed on the skin surface, followed by gradual disappearance and skin healing within 30 min (Fig. 2i). These data confirmed the minimally invasive puncture of MNA and indicated rapid skin healing after MNA puncture. In addition, in previous studies [11, 14], we demonstrated that microneedle materials have good biocompatibility in vitro and in vivo. Collectively, SOPL-customized MNAs can be used for skin puncture, laying the foundation for the regulation of the skin microenvironment for hair regeneration.

### Improvement of hair regeneration after MNA treatment

MNAs can be flexibly customized by SOPL and showed good skin puncture performance in this study. It has been reported that microneedle treatment regulates the skin microenvironment in the balding region and then induces hair regeneration [8, 9]. Herein, customized MNAs were further explored for precisely promoting hair regeneration in situ.

**Fig. 3:**
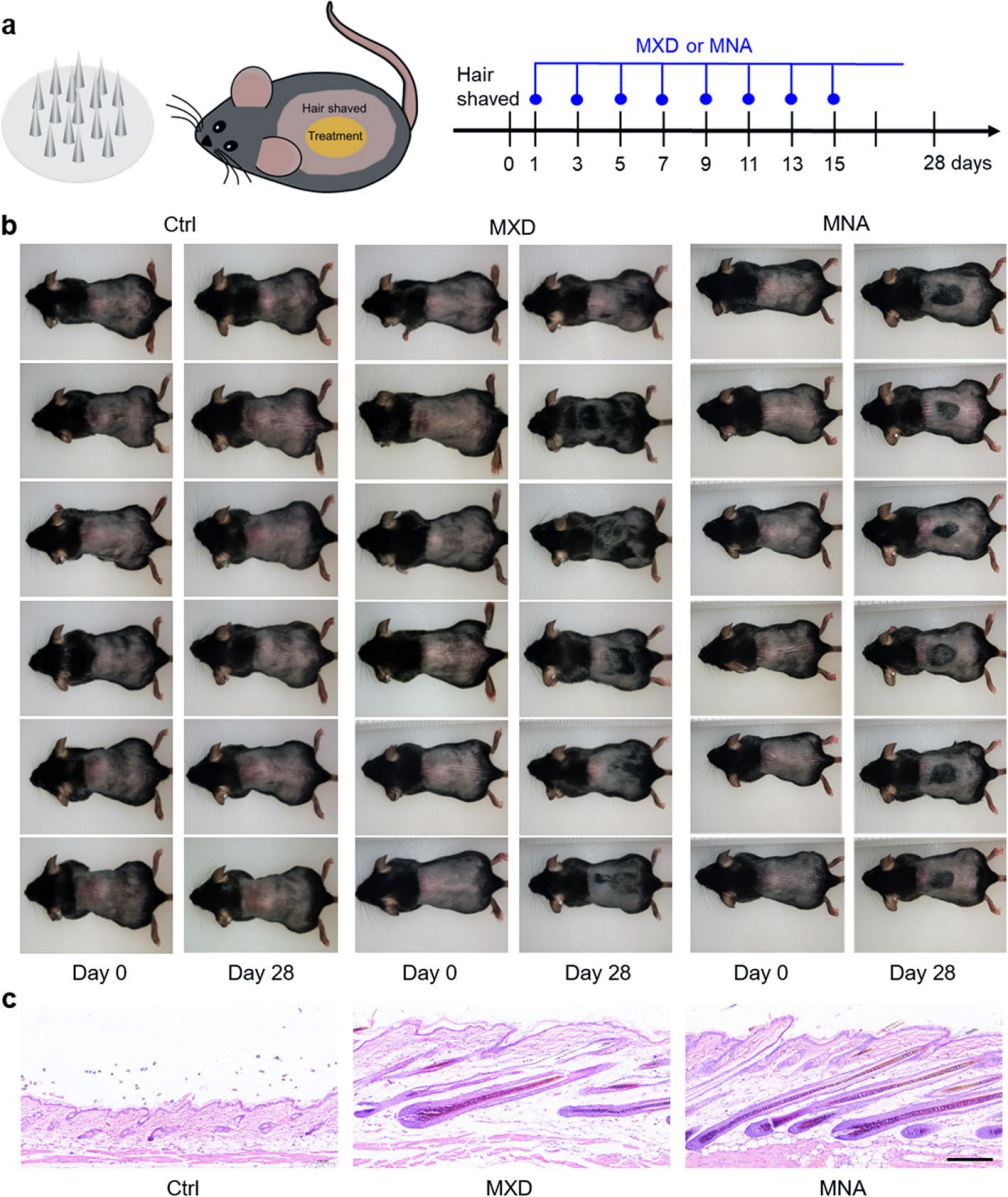
Hair regeneration induced by customized MNA. **a** Schematic diagram of MXD and MNA treatment. **b** Photos of mice taken on day 0 and day 28 showed that there was no hair regeneration in the control group, messy hair regeneration in the MXD group, and round-shaped hair regeneration in the MNA group, respectively (*n* = 6). **c** H&E staining on day 28 revealed that the hair follicles in the control group were in the telogen phase, and the majority of hair follicles in the MXD and MNA groups were in the anagen phase. (Scale bar: 200 μm)

C57BL/6 mice are one of the most commonly used animal models for the experimental evaluation of hair growth [19], as the hair follicles of mice aged 49 days enter the second telogen period which will last for 5 weeks [20]. In addition, female mice have a longer telogen period than the male [20, 21]. Therefore, female C57BL/6 mice aged approximately 7 weeks were selected for the study. In addition, mice with black spots on the skin, that is, abnormal hair cycles due to other reasons, were excluded from this study. The mice were treated as shown in the schematic diagram in Fig. 3a. The mice in the MNA group received treatment with a round-shaped MNA on the back for 5 s each time. Mice in the MXD group received a uniform round-shaped coating of MXD on the dorsal skin, while the mice in the control group received no treatment. Hair growth on day 28 is shown in Fig. 3b. This result demonstrated that there was no regenerated hair in the control group. This is because it normally takes approximately 5 weeks to enter the next anagen phase after mice enter the second round of postnatal telogen [20]. The MXD group showed a sparse and messy hair growth, which did not match the circular area of drug application. In contrast, the regenerated hair of the MNA group occurred in a circular area, which corresponded to the round shape of MNA. This indicated that treatment of MNA with the designed shape induced precise hair regeneration in situ in the target area. The H&E staining of treated skin on day 28 showed early active hair growth in both the MXD and MNA groups and resting telogen hair follicles in the control group (Fig. 3c), confirming that MNA treatment can induce early hair regeneration.

**Fig. 4:**
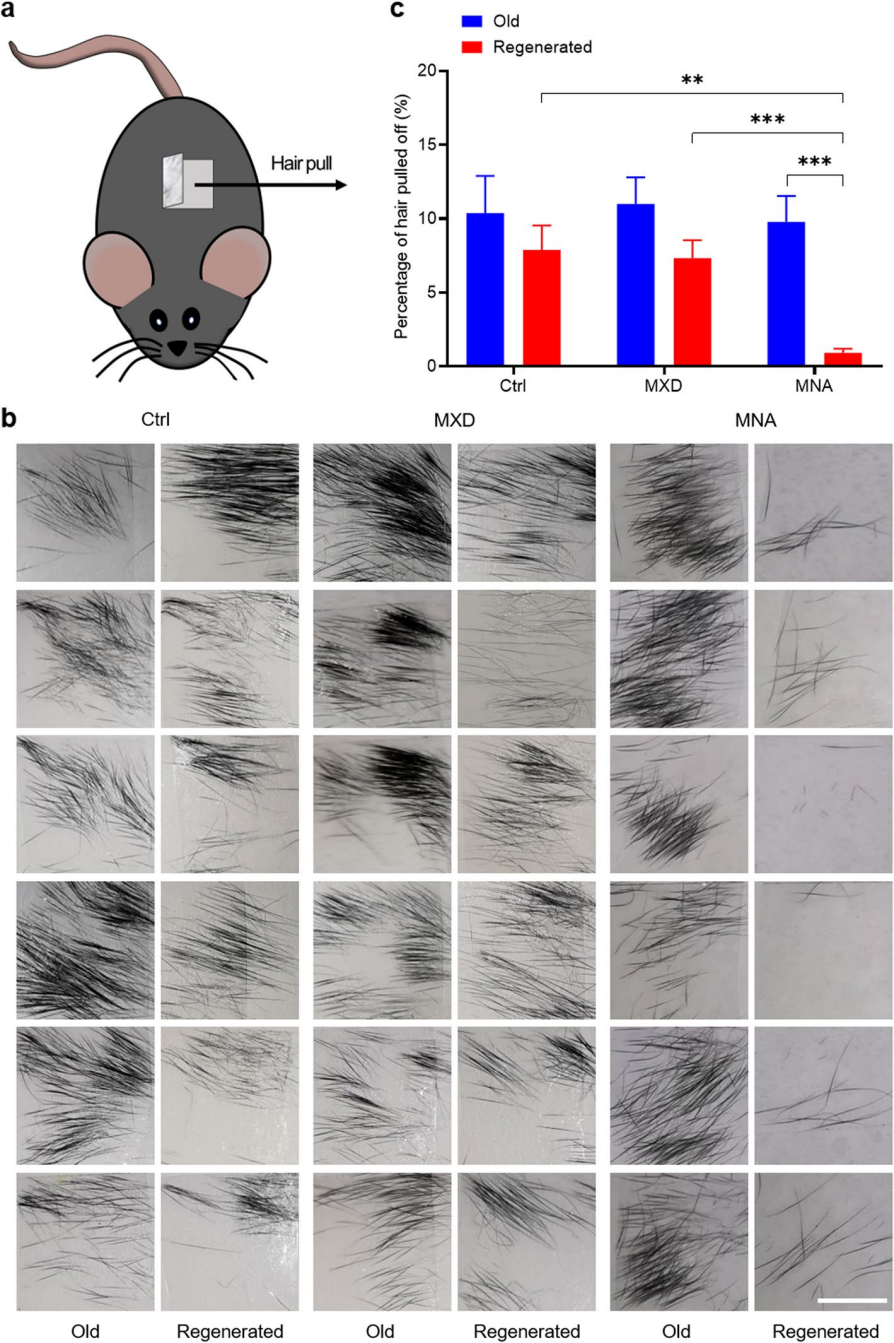
Improved quality of regenerated hair after MNA treatment. **a** Sketch of the hair pull test. **b** Hair pulled off on the tapes showed that shed hair was reduced in MNA-induced regenerated hair. (Scale bar: 0.5 cm). **c** The percentage of hair pulled off was obtained by calculating the gray value of the photos. *P < 0.05, **P < 0.01, ***P < 0.001, *n* = 6

To evaluate the quality of regenerated hair, a hair pull test was performed, as shown in Fig. 4a. The results showed no significant difference in the amount of old hair pulled off among all mice, and no significant difference between the amount of old and regenerated hair pulled off in the control and MXD groups (Fig. 4b). However, in the MNA group, reduced regenerated hair was found to be pulled off when compared with the old hair (Fig. 4b). By calculating the gray value of the photos in Fig. 4b, the percentage of hair pulled off was obtained, and the statistics confirmed the above results (Fig. 4c). This result indicated that the regenerated hair promoted by MNAs had improved quality.

### The potential mechanisms of MNA treatment in hair regeneration

**Fig. 5:**
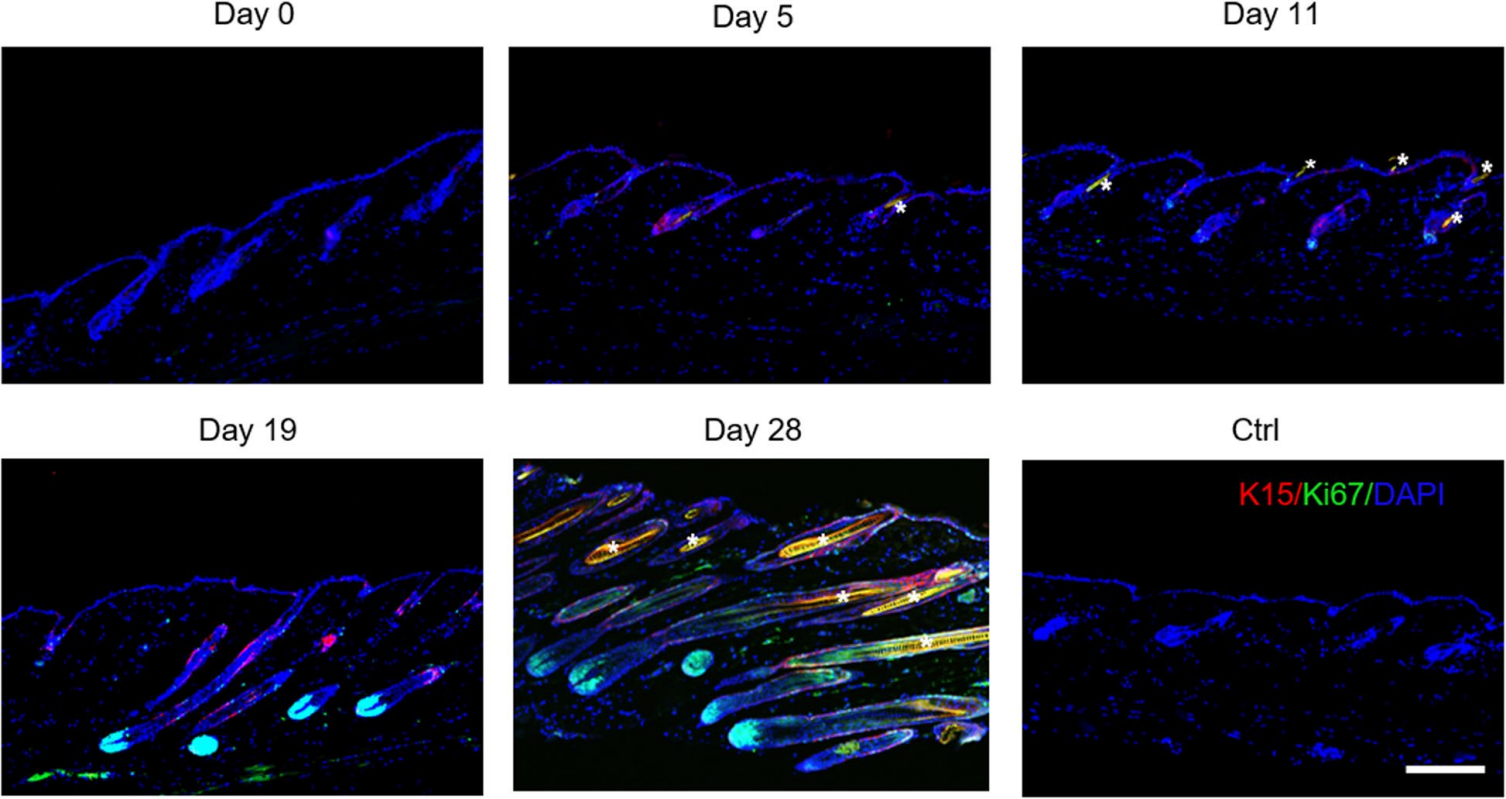
Hair follicle stem cells activated by MNA treatment. K15/Ki67 double-immunofluorescence staining on days 5, 11, 19 and 28 revealed that the hair follicle stem cells were gradually activated and proliferated after MNA treatment. (Scale bar: 100 μm). *Autofluorescence of hair shafts

After hair follicles are fully developed, they enter the cycle of catagen (degeneration), telogen (rest), and anagen (growth), during which hair follicle stem cell activation is accepted as a marker of hair follicle re-entry into anagen. Cytokeratin 15 (K15) is a common hair follicle stem cell marker and is widely used for hair regeneration study in mice [22–25]. K15/Ki67 double-immunofluorescence staining of the skins that received a 5-, 11-, 19-, and 28-day treatment showed that hair follicle stem cells were gradually activated by MNA treatment, which drove hair follicles to enter the anagen phase (Fig. 5). These data indicated that MNA treatment activated hair follicle stem cells and initiated their proliferation, realizing precise hair regeneration in situ.

**Fig. 6:**
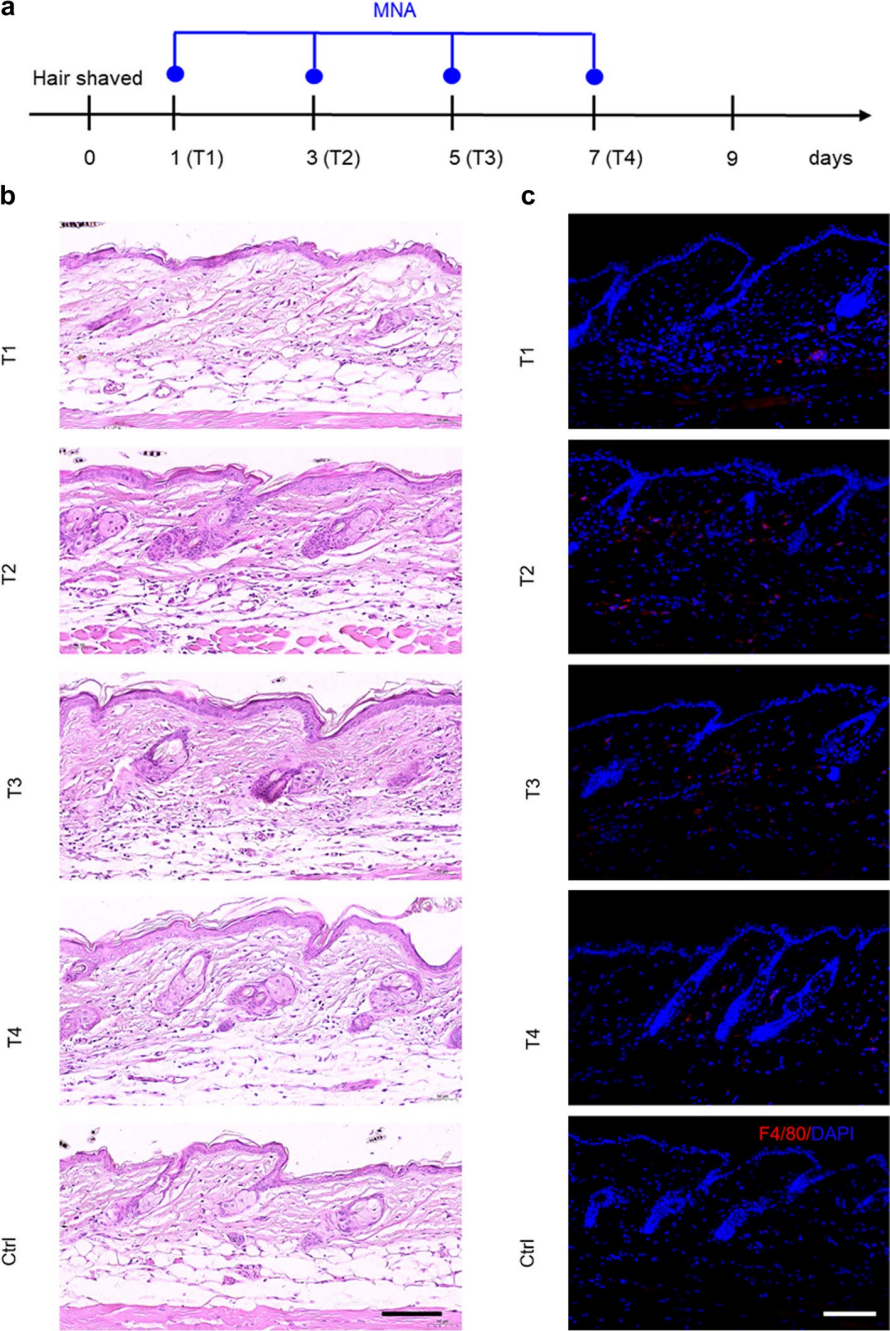
Macrophages recruited in situ by MNA treatment. **a** Schematic diagram of animal treatment. **b** H&E staining showed a small amount of inflammatory cell accumulation after 1, 2, 3 and 4 (T1, T2, T3, T4) MNA treatments (Scale bar: 100 μm). **c** Immunofluorescence staining demonstrated the accumulation of macrophages after MNA treatment (Scale bar: 100 μm)

Extrinsic injury can evoke intrinsic stimulation and subsequently initiate the physiological repair process [26]. MNA treatment has mechanical pressure and microinjury effects on the skin, but the detailed mechanism of MNA-induced hair regeneration in situ needs further exploration. Studies have reported that mechanical stretch [22] and hair plucking [27] induce hair regeneration by macrophage recruitment and subsequent secretion of growth factors or TNF-α. In addition, skin microinjuries [26] and wounds [28] also reportedly recruit macrophages to induce hair regeneration. Therefore, macrophages are accepted as the key cells in the regulation of hair regeneration triggered by mechanical stimulation and microinjury [22, 27, 28]. Here, the regulation of macrophages by MNA simulation was investigated. After a 1-, 3-, 5-, and 7-day MNA treatment (T1, T2, T3, T4), skin tissues were harvested for pathological staining (Fig. 6a). The H&E staining results preliminarily showed a small amount of inflammatory cell accumulation after MNA treatment (Fig. 6b). Immunofluorescence staining showed positive expression of F4/80, a macrophage surface marker, indicating macrophage accumulation after MNA treatment. Recruited macrophages were mainly distributed in the skin dermis (rich blood vessels) after first MNA treatment, and then macrophages accumulated around hair follicles after subsequent 2nd, 3rd, and 4th treatments (Fig. 6c). These data suggested that macrophages may be recruited from the blood circulation to the locally treated skin after MNA treatment.

**Fig. 7:**
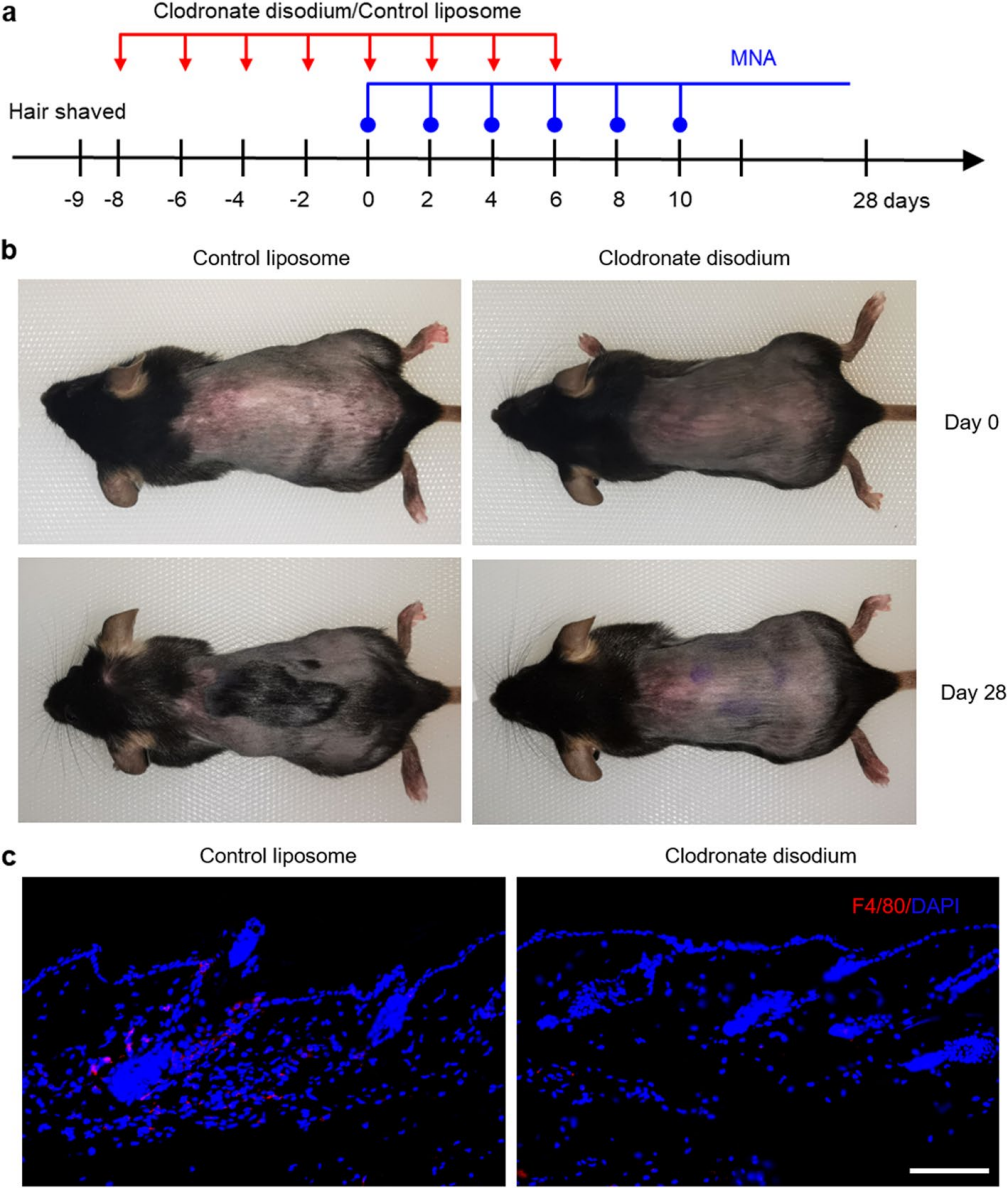
Abrogated MNA-induced hair regeneration after macrophage depletion. **a** Schematic diagram of animal treatment. **b** Representative photos of mice on day 28 showed that MNA-induced hair regeneration was impeded by intraperitoneal injection of clodronate disodium liposomes. **c** F4/80 immunofluorescence staining showed that MNA-induced macrophage recruitment was hindered after MNA treatment by intraperitoneal injection of clodronate disodium liposomes. (Scale bar: 100 μm)

To investigate whether macrophages play a functional role in MNA-induced hair regeneration, in vivo macrophages were depleted with clodronate disodium liposomes for reverse validation as reported by other studies [22, 27–30]. First, the physical properties of clodronate disodium liposomes and their ability to deplete macrophages in vitro were characterized (Fig. S1). Then, clodronate disodium liposomes were intraperitoneally injected to investigate the effect of MNA-induced hair regeneration after macrophage depletion (Fig. 7a). On day 28, hair regeneration occurred in the control liposome group but not in the clodronate disodium group (Fig. 7b). On day 8, F4/80 immunofluorescence staining showed that macrophages were found to accumulate in the control liposome group but not in the clodronate disodium group (Fig. 7c). In summary, MNA treatment could not recruit macrophages or induce hair regeneration after injection of clodronate disodium liposomes, confirming that macrophages are indispensable in MNA-induced hair regeneration.

**Fig. 8:**
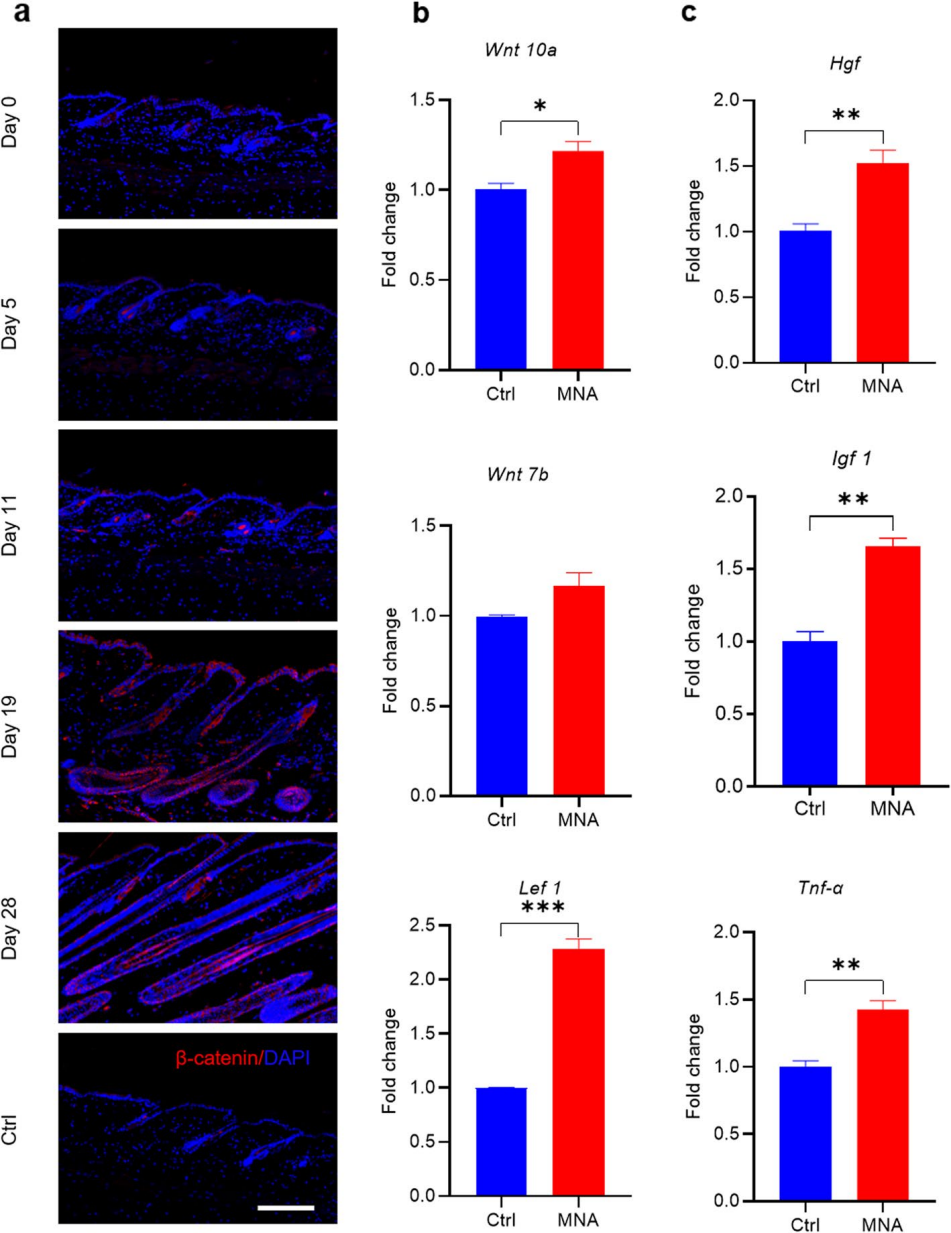
MNA-induced activation of the Wnt/β-catenin signaling pathway and the upregulation of related cytokines. **a** Immunofluorescence staining against β-catenin showed that MNA treatment increased the expression of β-catenin in hair follicles. (Scale bar: 100 μm). **b** RT-qPCR results showed the increased expression levels of *Wnt 10a* and *Lef 1* in the MNA group. *P < 0.05, **P < 0.01, ***P < 0.001, *n* = 3. **c** RT-qPCR results showed enhanced expression of *Hgf*, *Igf 1* and *Tnf-α* in the MNA group. *P < 0.05, **P < 0.01, ***P < 0.001, *n* = 3

Wnt/β-catenin signaling has been found to regulate stem cell-dependent tissue growth and this signaling pathway is essential in tissue regeneration [31, 32]. β-catenin is required in bulge stem cells for their proliferation [33], and activation of β-catenin in mouse hair follicle stem cells induces new hair growth [31]. Therefore, the Wnt/β-catenin signaling pathway is a key pathway for hair regeneration [27, 34, 35]. To investigate whether MNA treatment could activate the Wnt/β-catenin signaling pathway, skin tissues collected after a 5-, 11-, 19-, and 28-day MNA treatment were subjected to immunofluorescence staining against β-catenin. The results showed that the expression of β-catenin gradually increased with the cumulative stimulation of MNA, especially at the hair bulge, inner root sheath and outer root sheath of regenerated hair follicles (Fig. 8a). The expression of *Wnt 10a*, *Wnt 7b*, and *Lef 1*, the key components of the Wnt/β-catenin pathway [22], was then investigated by real-time qPCR (RT-qPCR) after a 19-day MNA treatment. The results showed enhanced expression of *Wnt 10a, Lef 1* and *Wnt 7b* (Fig. 8b). These results indicated that the Wnt/β-catenin signaling pathway was activated during the process of MNA-induced hair regeneration.

Some studies have reported that recruited macrophages can activate hair follicle stem cells to induce hair regeneration by releasing HGF, IGF 1 and TNF-α [22, 27, 28, 36], which was subsequently investigated in this study. The results of RT-qPCR after three MNA treatments showed that the expression levels of *Hgf*, *Igf 1* and *Tnf-α* in the MNA group were significantly higher than those in the control group (Fig. 8c). This result suggested that MNA treatment could upregulate the expression of *Hgf*, *Igf 1* and *Tnf-α*, which may be involved in MNA-induced hair regeneration.

## Discussion

Alopecia is a common dermatological disorder and has negative psychological impacts on patients [37]. Currently, precise hair regeneration is urgently needed for alopecia patients since they suffer from different balding conditions. However, common pharmaceuticals, such as MXD and finasteride, can hardly realize personalized hair regeneration. Therefore, how to precisely control hair regeneration remains a challenge. Currently, MNA treatment alone has been reported to have the potential to promote hair regrowth. Herein, we demonstrate a strategy using customized MNAs to precisely control hair regeneration in situ. In the mouse model, the round-shaped MNA induced hair regrowth with a round shape at the site of action. MNA treatment showed improvement of hair quality when compared with MXD, indicating that healthier hair follicles may be obtained after MNA treatment. Collectively, this work provides a novel method to precisely control hair regeneration using customized MNAs, which would meet the needs of different balding conditions and advance personalized treatments for hair loss.

Customized MNAs hold great application prospects in the personalized treatment of hair loss. However, the clinical translation of customized MNA is restricted by the fabrication methods. The rapid customization of the MNA can hardly be achieved by the commonly used micromolding methods. 3D printing technology provides a powerful manufacturing technology for fabricating personalized structures [38, 39]. However, the 3D printing technologies for preparing MNAs are usually based on layer-by-layer printing, which is very time-consuming. The obtained MNA with a layer-by-layer structure affects the structural integrity and mechanical properties of the MNA. Therefore, present 3D printing technologies need to be improved in terms of the manufacturing speed and the MNA quality. Recently, we developed an advanced 3D printing technology (SOPL) for the fast customization of high-quality MNA [11, 14], which holds great promise for the clinical translation of MNA. In the SOPL process, the beam is modulated into customized patterned light by DMD, and then the patterned light is projected into the photosensitive resin to induce precise “static” photopolymerization of the monomer solution according to the spatial distribution of the light intensity, without the aid of a mechanical motion device for layer-by-layer printing. As a result, high-quality MNAs, without layer-by-layer structure common in the 3D-printed products, can be fast customized within seconds. It is demonstrated that SOPL technology is the fastest method to customize high-quality MNAs among the existing methods, which significantly saves manufacturing time and cost. In addition, SOPL technology can be used to fabricate large-size MNAs by enlarging the size of DMD chips. Personalized MNAs can be customized to fit the skin surface using a resilient resin. Therefore, the SOPL technique provides technical support for the rapid customization of MNA and its application for precisely controlling hair regeneration, showing great promise for clinical translation.

Currently, microneedling is widely used in dermatology, including scars, acne, melasma, photodamage, skin rejuvenation, hyperhidrosis, alopecia, etc. [40]. It treats a variety of skin conditions by inducing controlled inflammatory/healing reactions and remodeling collagen by promoting the release of growth factors [41]. The preliminary results revealed that microneedling has promising therapeutic benefits for hair loss [8], but the mechanism of microneedling monotherapy still needs further investigation. In this study, we first found that MNAs induced macrophage-dependent hair regeneration. Customized MNAs recruited macrophages in situ and then initiated the proliferation of hair follicle stem cells for hair regeneration, which was abrogated after macrophage depletion. Macrophages are indispensable in MNA-induced hair regeneration, which may be a novel mechanism of MNA-mediated hair regeneration. In detail, the potential underlying mechanism is that MNAs stimulate skin to produce chemokine (C-C motif) ligand 2 (CCL2) by repeated microinjury and mechanical stimulation [22, 27]. Then macrophages are recruited by the chemokines and may further undergo polarization. Later, the regenerative phenotype of macrophages release cytokines, such as HGF, IGF-1, and TNF-α, and then activate proliferation of hair follicle stem cells by upregulating the Wnt/β-catenin pathway [22, 27]. In addition, macrophages play critical roles in regulating multiple phases of tissue repair [42]. Collectively, these results indicate that MNA treatment with the function of recruiting macrophages holds great promise for alopecia and provides a new strategy for regenerative medicine.

**Fig. 9:**
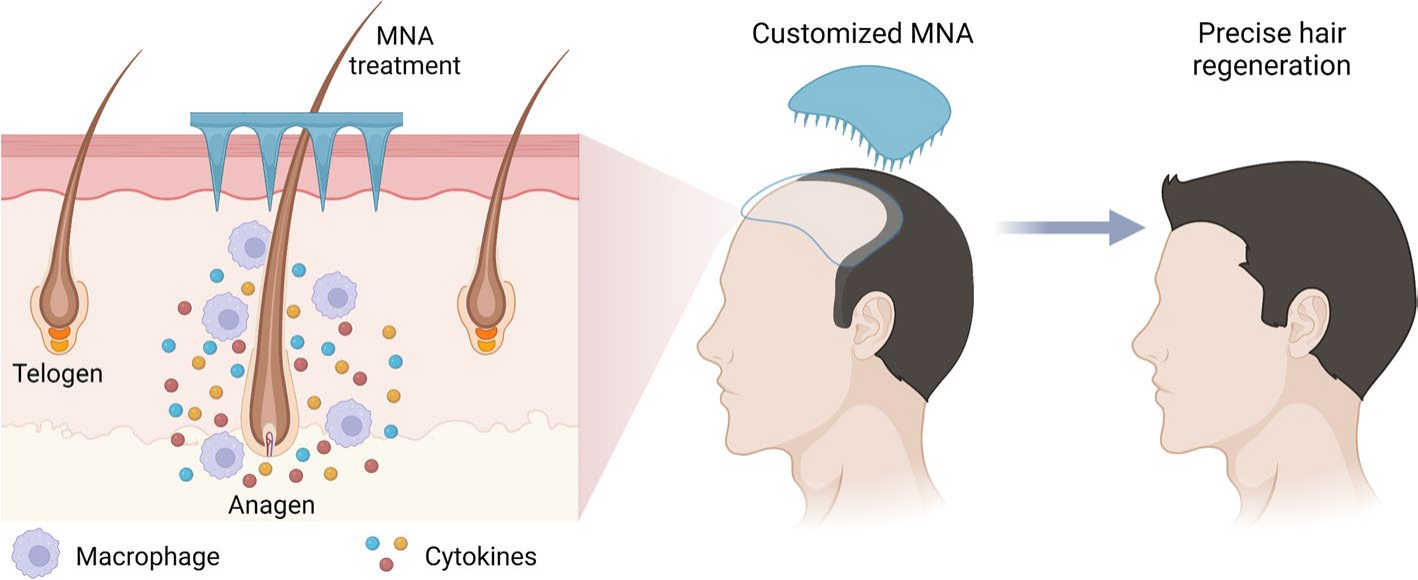
Improving hair regeneration by customized microneedle arrays by recruiting macrophages in situ. The MNA treatment could induce hair regrowth in a defined area corresponding to the customized shape of the MNA. This result indicated that MNA treatment could recruit macrophages in situ and then initiate the proliferation of hair follicle stem cells, thereby improving hair regeneration. Moreover, the relative cytokines (*Hgf*, *Igf-1*, and *Tnf-α*) were also upregulated in the treated skin. This method holds great potential for the personalized treatment of hair loss, which could lead to the development of future regenerative medicine. Created with BioRender.com.

In summary, this work demonstrated the potential of 3D-printed customized MNA to precisely control hair regeneration by recruiting macrophages in situ (Fig. 9). The MNA with a customized shape is quickly fabricated by the SOPL technology within seconds. The MNA treatment could induce hair regrowth in a desired area corresponding to the customized shape of the MNA. We also revealed that macrophages played an important role in MNA-induced hair regeneration. In detail, MNA treatment could recruit macrophages in situ and then initiate the proliferation of hair follicle stem cells, thereby improving hair regeneration. Moreover, the activation of Wnt/β-catenin was observed in the treated hair follicles. The expression of *Hgf*, *Igf 1* and *Tnf-α* was also upregulated in the treated area. This study provides a new strategy for the personalized treatment of hair loss, which could also lead to the development of future regenerative medicine. In addition, MNAs can also be used to promote the transdermal delivery of drugs. Combined with the drug delivery, we believe that MNA treatment will inspire new approaches for alopecia. Nevertheless, our strategy still has limitations. MNAs, like other pharmaceutical treatments, may have no therapeutic effect on cicatricial alopecia in which the structure of hair follicles has been damaged.

## Materials and methods

### Materials

Photosensitive resin was purchased from Ausbond (Shenzhen, China). Hair removing cream of VEET was obtained from Reckitt Benckiser Plc. (Wuhan, China). Isoflurane was purchased from Shenzhen Reward Life Technology Co., Ltd. (Shenzhen, China). MXD liniment (2%) was purchased from Sichuan Medco Huakang Pharmaceutical Co., LTD. (Sichuan, China). DAPI-containing antifluorescence quenching tablets were obtained from Beyotime Biotechnology (Shanghai, China). Cell-Counting Kit-8 was purchased from Dalian Meilunbio Co. Limited. (Dalian, China). Clodronate disodium was obtained from ShangHai YuanYe Biotechnology Co., Ltd. (Shanghai, China).

The following antibodies were used for immunostaining: primary antibody: anti-β-Catenin (Wanlei bio, WL 0962a, 1:200), anti-CK15 (HuaBio, ET1609-54, 1:100), anti-Ki67 (ThermoFisher Scientific, 14-5698-80, 1:200), and anti-F4/80 (Servicebio, GB113373, 1:500). Secondary antibodies: fluorescence conjugates with rhodamine (ZSGB-BIO, 1:100), Cy3 (Servicebio, 1: 200), and FITC (ZSGB-BIO, 1:100).

### Animals

Female C57BL/6 mice (7 weeks old) were provided by Chengdu Dossy Experimental Animals Co.,Ltd.

### 3D printing of customized MNAs

The MNAs were fabricated using SOPL, a 3D printing technology previously developed by our group [11]. The SOPL device consists of a light source, DMD chip, optical lens, and reservoir. For 3D printing of customized MNAs, we first drew the printing picture of the MNA using Adobe Photoshop CC 2019 software. The printing picture of a microneedle is a white circle, and multiple circles arranged in an array can be used to print a MNA, such as multiple white circles distributed in a circular arrangement to fabricate a round-shaped MNA. Next, the printing picture was imported into DLP LightCrafter 4500 control software, and DMD modulated the light beam into a customized light pattern according to the printing picture. Then, the patterned light was projected into the reservoir, forming a special spatial distribution of light intensity in the monomer solution and inducing the spatial polymerization of monomer solutions to form the MNA.

MNA was printed on silanized modified glass slides to prevent shedding, and washed with absolute ethanol 3 times (2 min each time) to completely remove the residual resin solution on the surface of MNA. After drying, they were exposed to ultraviolet (UV) -Vis radiation at 405 nm for 3 min to polymerize the remaining uncured resin for better mechanical properties. Then, they were soaked in 75% ethanol and 1 × phosphate-buffered saline (PBS) for 24 h to meet the requirements of biocompatibility. Finally, the MNA was dried and stored at room temperature for later use. The morphologies of the customized MNA were observed by camera and SEM (JSM-7500 F) at 15 kV.

### Simulation of microneedle formation process

To visualize the light propagation and intensity distribution of microneedle formation process, finite element simulation was implemented using the electromagnetic wave beam envelope module in COMSOL Multiphysics software. The digital light propagation direction was set as the z direction, and the position of the liquid resin surface was z = 0. In addition, the digital light with uniformly distributed light intensity propagates along the z direction. The original intensity at *z =* 0 was set to be constant, and the attenuation coefficients along the needle growing direction were set as ${C}_{0}=0.9627$, ${C}_{1}=0$, and ${C}_{2}=0.002878$, as mentioned in the literature [17]. Owing to the conical surface similar to the parabolic curve throughout the microneedle forming process, a simplified 2D geometric model with two parabolic edges imitating the cone-like microneedle at the center with designed height was constructed in COMSOL, and the base diameter was set to be 100 μm. Obtained from a universal database, the refractive indexes of liquid resin and solidified resin are 1.45 and 1.55, respectively, and the refractive index of the glass substrate is 1.5. The geometrical area was then triangularly meshed with a maximum element size of wavelength λ, and the total model was discretized cubically. All the simulated intensity data have been normalized into a range of 0 to 1.

### Evaluation of mechanical properties

Compression tests of a single microneedle and MNA were performed to assess the mechanical properties using dynamic mechanical analysis (DMA) (Q800 TA Instruments). Taking a single microneedle as an example, place the microneedle vertically on the test bench and gently move a metal probe to contact the tip of the MN. The metal probe was then set to move vertically downwards with a compressive force of 2.5 N/min. The compression process and morphologies of the microneedle before and after compression were captured by a camera. TA Instruments Universal Analysis 2000 (DMA software) recorded the relationship between compression displacement and force. The turning point of the curve is the point at which the microneedle undergoes severe deformation.

### Investigation of puncture performance and skin healing

The dorsal skin of the mice was shaved and depilated and then cleaned with normal saline. MNA was applied to the dorsal skin of the mice for 5 s, and then the skin was sampled immediately and fixed with 4% paraformaldehyde for 24 h, followed by rinsing, dehydrating, waxing, sectioning (5 μm), and H&E staining. Spectralis OCT (Heidelberg) was used for skin puncture imaging of the MNA. The MNA was pressed into ex vivo mouse skin of approximately 1 × 1 cm [2], and then the sample was mounted on a sample holder for photography. The parameters of the imaging system were as follows: a superluminescent diode light source with a wavelength of 870 nm, 6 μm lateral resolution and 5 μm vertical resolution. Then, the healing process of micropores on the skin was observed at 0, 5, 10, 15, 20 and 30 min after MNA application and photographically recorded with a camera.

### Customized MNAs for precisely controlling hair regeneration in situ

The mice were shaved with an electric razor and randomly divided into three groups: the control group, MXD group and MNA group. The control group was not treated. The MXD and MNA groups were treated every other day until the dorsal skin began to darken, revealing that the hair follicles entered anagen phase. For the MNA group, a round-shaped MNA was applied to the same circular area on the back each time and pressed for 5 s. For the MXD group, 20 µL 2% MXD (0.4 µg) was smeared evenly to the circle in the same position with a cotton swab each time. These circular areas were marked with a skin marker to ensure the same location for each application. At 28 days, hair regeneration was observed by photographing, and the dorsal skin of the treated site was harvested for H&E staining.

### Quality assessment of regenerated hair

Hair pull test was used to evaluate the quality of the regenerated hair [43, 44]. When the regenerated hair entered the telogen phase, the regenerated hair and the old hair in the unshaved area were pulled with transparent adhesive tape of approximately 1 × 1 cm. Then the tapes with hair pulled off were pasted to the A4 paper for photographing. ImageJ software was used to calculate the gray value of the photos to indirectly analyse the amount of hair pulled off. The total gray value represented the total gray value in the area without hair, and blank tape of the same size was set as the control, indicating that no hair was pulled off. The formula is shown as follows:

$$\text{The percentage of hair pulled off = [Total gray value (blank tape) - Total gray value (experimental group)]/Total gray value (blank tape) * }100\%$$

### Animal studies on the mechanisms of MNA-induced hair regeneration

To investigate the activation of hair follicle stem cells during MNA treatment, skin tissues sampled on days 5, 11, 19, and 28 after MNA treatment, were used for K15/Ki67 double-immunofluorescence staining.

To investigate whether MNA treatment recruits macrophages, skin tissues were harvested for staining after 1, 2, 3 and 4 rounds of MNA treatment, respectively. For in vivo macrophage depletion, mice were intraperitoneally injected with 200 µl of clodronate disodium liposomes or control liposomes every other day for a total of 8 times, and MNA treatment was started before the 5th injection. On the 8th day, some mice were sampled for immunofluorescence staining, and the other mice continued to be treated with MNA until the skin darkened.

To investigate the molecular mechanisms of hair regeneration induced by MNA, mice were shaved and divided into control and MNA groups. Mice were then treated with MNA as previously mentioned. Skin tissues were harvested on days 5, 11, 19, and 28 after treatment and equally divided into two parts. One part was used for immunofluorescence staining and the other part was used for RT‒qPCR experiments, to investigate the Wnt/β-catenin signaling pathway and the related cytokines.

### Immunostaining

For immunofluorescence analysis, the collected skins were fixed with 4% paraformaldehyde for 24 h, followed by rinsing, dehydrating, waxing, sectioning (5 μm), and staining. The staining procedures were as follows: paraffin sections of skin tissues were deparaffinized in water, and microwave antigen retrieval was performed with 0.01 M citrate buffer (pH 6.0) for 5 min with high fire, 3 min with ceasefire, 5 min with medium fire, and approximately 20 min with natural cooling to room temperature and then washed with PBS (3 times, 5 min each). Sections were blocked with goat serum at 37 ℃ and incubated for 20 min. The primary antibody was added and incubated overnight at 4 ℃. The next day, the tissues were rewarmed at room temperature for 20 min and washed with PBS (3 times, 5 min each). Fluorescent secondary antibodies were added, and incubated for 1.5 h at 37 ℃ in the dark, and washed with PBS (3 times, 10 min). The sections were sealed with DAPI-containing antifluorescence quenching tablets and observed under a fluorescence microscope.

### RT‒qPCR

First, RNA in skin tissue was extracted according to the manufacturer’s recommendations of the total RNA isolation kit (FOREGENE). Second, the extracted total RNA was used as template RNA for reverse transcription according to the RT EasyTM II instruction manual (FOREGENE). Finally, the mRNA expression levels were analysed by RT-qPCR with MonAmp™ SYBR® Green qPCR Mix (Monad Biotech). Target gene-specific primer sequences and suitable probes were designed by the National Center for Biotechnology Information website. Relative expression was normalized to that of *GAPDH* for each sample. The primers for RT-qPCR amplification are summarized in Table S1.

### Statistical analysis

All statistical analyses were performed using GraphPad Prism 8 and statistical significance was determined using Student’s t test or ANOVA (*P < 0.05, **P < 0.01, ***P < 0.001). Each experiment was performed at least three times and *n* represents the number of independent biological replicates.

## Supplementary Information

**Additional file 1: Supplementary Fig. 1.** Characterization of self-prepared clodronate disodium liposomes. **Supplementary Table 1.** Primers for Real-Time qPCR analysis.

## Data Availability

The data of this study are available from the corresponding author upon reasonable request.

## Abbreviations

MNAs: Microneedle arrays
MXD: Minoxidil
SOPL: Static optical projection lithography
*Hgf*: Hepatocyte growth factor
*Igf-1*: Insulin-like growth factor 1
*Tnf-α*: Tumor necrosis factor-α
DMD: Digital micromirror device
*H*: Height
SEM: Scanning electron microscope
H&E: Hematoxylin-eosin
OCT: Optical coherence tomography
K15: Cytokeratin 15
PBS: Phosphate-buffered saline
DMA: Dynamic mechanical analysis
RT-qPCR: Real-time qPCR
