# Supplementary material for: 3D printing of microneedle arrays for hair regeneration in a controllable region
Source: Mol Biomed. 2023 Jan 5;4:1. doi: 10.1186/s43556-022-00102-2 (PMC9816368; doi:10.1186/s43556-022-00102-2)
Supplement: Supplementary file 1 — Additional file 1: Supplementary Fig. 1. Characterization of self-prepared clodronate disodium liposomes. Supplementary Table 1. Primers for Real-Time qPCR analysis. [file 43556_2022_102_MOESM1_ESM.docx]

Supplementary Information for

## 3D printing of microneedle arrays for hair regeneration in a controllable region

Rong Li^1†^, Xin Yuan^1,2†^, Li Zhang^1^, Xuebing Jiang^1^, Li Li^1^, Yi Zhang^1^, Linghong Guo^3,4^, Xide Dai^1^, Hao Cheng^1,5^, Xian Jiang^3,4^, Maling Gou^1*^

^1^ State Key Laboratory of Biotherapy and Cancer Center, West China Hospital, Sichuan University, Chengdu, 610041, China.

^2^ Department of Plastic and Burn Surgery, West China Hospital, Sichuan University, Chengdu, 610041, China.

^3^ Department of Dermatology, West China Hospital, Sichuan University, Chengdu 610041, China.

^4^ Laboratory of Dermatology, Clinical Institute of Inflammation and Immunology (CIII), Frontiers Science Center for Disease-related Molecular Network, West China Hospital, Sichuan University, Chengdu 610041, China.

^5^ Huahang Microcreate Technology Co., Ltd, Chengdu 610042, China.

* Email: [goumaling@scu.edu.cn](mailto:goumaling@scu.edu.cn).


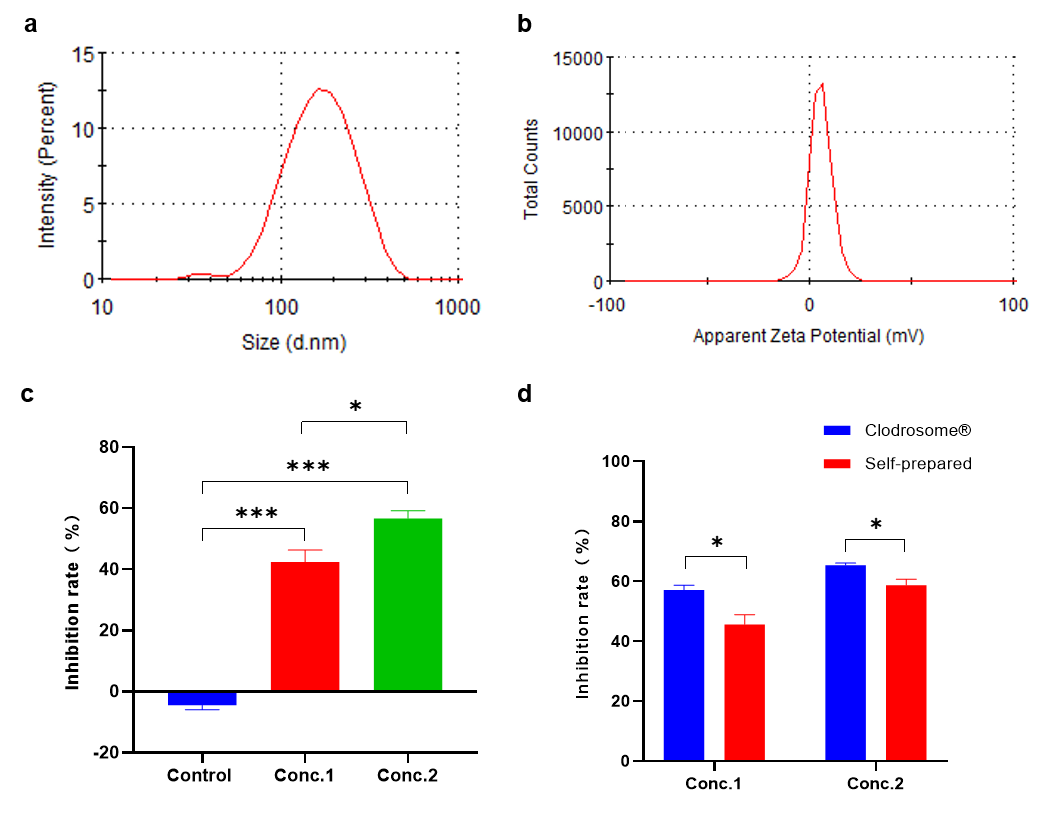
**Supplementary Fig.1 Characterization of self-prepared** **clodronate disodium liposomes.** (**a**) Particle size was about 150 nm. (**b**) ζ potential was 0.481 mV. (**c**) CCK-8 results showed that clodronate disodium liposomes inhibited macrophage proliferation, while empty liposomes did not. (**d**) Self-prepared clodronate disodium liposomes were slightly less effective than Clodrosome® in inhibiting macrophage proliferation, but could be used to deplete macrophages. *P < 0.05, n=4.

**Supplementary Table 1** Primers for Real-Time qPCR analysis.

| Genes | Forward | Reverse |
| --- | --- | --- |
| *Wnt10a* | TCCTGTTCTTCCTACTGCTGCT | AGGATGTCGTTGGGTGCT |
| *Wnt7b* | CATTCCTGAACCCAGCCGA | ACGGATGACAATGCTCTGTAAG |
| *Lef1* | TCCTGAAATCCCCACCTTCT | TGGGATAAACAGGCTGACCT |
| *Hgf* | AACAGGGGCTTTACGTTCACT | CGTCCCTTTATAGCTGCCTCC |
| *Igf 1* | AAATCAGCAGCCTTCCAACTC | GCACTTCCTCTACTTGTGTTCTT |
| *Tnf-α* | ACCCTCACACTCACAAACCA | ACCCTGAGCCATAATCCCCT |
| *GAPDH* | TGGCCTTCCGTGTTCCTAC | GAGTTGCTGTTGAAGTCGCA |
